# Patient apprehensions about the use of artificial intelligence in healthcare

**DOI:** 10.1038/s41746-021-00509-1

**Published:** 2021-09-21

**Authors:** Jordan P. Richardson, Cambray Smith, Susan Curtis, Sara Watson, Xuan Zhu, Barbara Barry, Richard R. Sharp

**Affiliations:** 1 Biomedical Ethics Research Program, Mayo Clinic Rochester, Rocheste, MN 55905-0002 USA; 2 Kern Center for the Science of Healthcare Delivery, Mayo Clinic Rochester, Rocheste, MN 55905-0002 USA

**Keywords:** Medical ethics, Health policy, Translational research

## Abstract

While there is significant enthusiasm in the medical community about the use of artificial intelligence (AI) technologies in healthcare, few research studies have sought to assess patient perspectives on these technologies. We conducted 15 focus groups examining patient views of diverse applications of AI in healthcare. Our results indicate that patients have multiple concerns, including concerns related to the safety of AI, threats to patient choice, potential increases in healthcare costs, data-source bias, and data security. We also found that patient acceptance of AI is contingent on mitigating these possible harms. Our results highlight an array of patient concerns that may limit enthusiasm for applications of AI in healthcare. Proactively addressing these concerns is critical for the flourishing of ethical innovation and ensuring the long-term success of AI applications in healthcare.

## Introduction

Artificial Intelligence (AI), the ability of computers to perform tasks typically associated with human intelligence^1^, has the capacity to impact millions of patients by changing the way medicine is practiced. Enthusiasm for applications of AI in healthcare has continued to grow, with early successes involving ChatBots, diagnostic tools, and radiological image analysis^2–5^. While there is considerable excitement about these emerging technologies, prospective analyses of how AI technologies might be implemented responsibly into clinical practice has been limited. Importantly, to date there has been very little engagement with patients who will be impacted by applications of AI in healthcare. This is troubling since patient concerns about AI could be a significant barrier to the dissemination and use of these tools. Studies of nonmedical applications of AI have shown that the public tends to view nonmedical AI in highly variable ways^6^, with factors such as media coverage and early experiences playing key roles in shaping public opinion. These considerations highlight the importance of patient engagement to ensure that these technologies are integrated into healthcare in a manner that fosters public trust^7,8^ and mitigates widespread patient concerns that might result in another “AI Winter”^9^.

Moreover, since patients are the intended beneficiaries of many of these AI innovations, more carefully characterizing their needs, values, and priorities is important for ensuring that these advances are not just well-received but are developed and implemented in an ethical way that improves patient care. Even in situations where patients do not interface directly with AI technologies, patients still bear the largest risk should implementation be done incorrectly or unethically^10^. To the extent that patients will be asked to accept the potential risks associated with novel applications of AI in healthcare, there is an ethical obligation to ensure that patient values and needs are incorporated into implementation plans. As in other areas of medical innovation, proactive patient engagement is an essential component of implementing healthcare AI in an ethical manner^11^.

Applications of AI in medicine take advantage of unprecedented volumes of clinical data and computing power to inform evidence-based decision making^3^. This raises new ethical questions related to the transparency of data use, accountability for data stewardship, and potential inequities in the deployment of AI^12^. Currently, very little research has been done characterizing patient and other stakeholder perspectives on applications of AI in healthcare. Additionally, the few studies that have assessed patient perspectives have focused on a narrow array of AI tools, which limits their utility as a guide in anticipating patient engagement with other AI applications in healthcare^13,14^. While engaging patients around specific applications of AI is a crucial step in the research and development process, engagement at this level of specificity does not facilitate analysis of broader public perspectives on AI and its application in healthcare, which is much needed for health policy development, innovation priority setting, and implementation design.

The aim of the research study we report was to understand how patients view the use of AI in their healthcare. To clarify sources of patient excitement and concern about healthcare AI, we used focus groups and case-study discussions to characterize the range of patient opinions about emerging AI applications in healthcare. This approach allowed us to make AI technologies more accessible to patients, while also engaging across diverse cases to promote broader reflection on the potential technological, societal, and medical impacts of AI. The results we report highlight a range of patient concerns about applications of AI in healthcare. We hope AI developers and healthcare institutions seeking to deploy new AI technologies find these results useful as they consider how best to integrate AI technologies into healthcare and create governance structures that promote patient safety and foster the trust of the patients they serve.

## Results

We conducted 15 focus groups with 87 participants between November 2019 and February 2020. Each focus group had between three and seven participants and lasted 90 min. Approximately half of our participants were female (49.4%) and the average age of participants was 53.5 years old. A majority of participants were white (93.1%) and non-Hispanic/Latino (94.3%). Most participants had an education level higher than a high school degree (87.3%). Approximately one in five participants had experience working in technology or computer science (19.5%) for an average of 17.6 years. Nearly half of our participants had experience working in healthcare or health science (44.8%) for an average of 17.1 years. No participants reported any prior experience with AI impacting their healthcare. A detailed description of these and other participant characteristics is presented in Table 1.

**Table 1:** Characteristics of 87 patients who participated in focus groups examining attitudes about the use of AI in healthcare.

	*N* (%)
Sex	
Male	44 (50.6)
Female	43 (49.4)
Ethnicity	
Not Hispanic	82 (94.3)
Choose not to Answer	5 (5.7)
Race (select all that apply)	
White	81 (93.1)
Black or African American	3 (3.4)
American Indian or Alaska Native	2 (2.3)
Asian	1 (1.1)
Choose not to Answer	2 (2.3)
Age (Mean 53.5 years; Range 18–91)	
Age 18–40	24 (27.6)
Age 41–60	29 (33.3)
Age 61+	34 (39.1)
Education	
Less than HS Grad	1 (1.1)
Grade 12 or GED	10 (11.5)
College 1–3 years	31 (35.6)
College 4 years or more	23 (26.4)
Graduate school	22 (25.3)

In what follows, we describe several major themes that emerged during focus-group discussions of healthcare AI. These themes reflect multiple sources of patient concern and excitement about applications of AI in medicine. We found that, while patients are generally enthusiastic about the possibility of AI improving their care, they are also concerned about the safety and oversight of healthcare AI. We describe these concerns about AI safety, the importance of patient choice, concerns about rising healthcare costs, questions about data quality, and view of the security of AI systems below.

### Participants were excited about healthcare AI but wanted assurances about safety

In general, participants reported enthusiasm about the ability of AI to be a positive force in medicine. They felt healthcare AI was compatible with the goals of medicine: to heal as many patients as possible. Participants were supportive of developing AI tools for a variety of different healthcare applications.

*I feel good about it. I think it has the ability to be better. I mean, it’s not a human. It’s got more data, so probably. … [I]t probably has more intelligence; it just has more information to work with to try to come up with a proper diagnosis. … I don’t think you will cure a lot of diseases without that advanced intellect. Obviously, we’ve come a long way with the human brain, but we could probably go a lot farther and speed the process with AI. (FG14)*.

Our participants also reported being aware that healthcare AI was still an emerging technology. As such, participants felt that it still had potential to be used in many different creative and positive ways.

This was often expressed as a sentiment of hopefulness, coupled with an acknowledgment that these benefits could only be realized through thoughtful implementation.

*I feel like the future of AI just depends on how we choose to use it. The impact will be what we choose it to be. … Because it’s moldable, it’s not going to do anything that we don’t allow it to do. (FG7).*

Participants urged caution in developing and implementing AI tools. They reported a need for a careful transition period to ensure that any AI tool used in their care is well-tested and accurate.

*So when this intelligence is built we have to test it, right? We have to test it to make sure that it’s helping correctly, and that to me represents a big challenge and one we don’t wanna jump into and see what happens. We’ve gotta be very careful there. (FG4).*

Participants also called for oversight and regulatory protections against potential harms. While participants often could not articulate what these regulations should include or who should enact them, they felt additional protections were necessary for AI tools.

*It’s gonna do what it’s gonna do. I think that I look forward with excitement… but I agree that there’s definitely some flaws, and there definitely needs to be some markers in there, at least right now, that can also protect people. But it’s going to be positive if we can get those safe markers in. (FG11).*

### Patients expect their clinicians to ensure AI safety

Participants reported that they felt their clinicians should act as a safeguard to buffer patients from the potential harms that might result from mistakes made by healthcare AI. One way this was commonly expressed was in terms of their healthcare providers retaining final discretion over treatment plans and maintaining responsibility for patient care.

*I believe the doctor always has the responsibility to be checking for you, and you’re his responsibility, you know? The AI is not responsible; that’s just a tool. (FG13).*

Other participants were comfortable extending more authority to AI tools, but still calling for their providers to provide “checks and balances” or “second opinions” on recommendations generated by healthcare AI. Most participants felt strongly that an AI algorithm should not have the ability to act autonomously in a clinical setting, stressing that both treatment decisions and the monitoring of ongoing care should be done by a human provider.

*I’d be okay with them telling a doctor what to do, but I don’t know that I’d want a machine doing the treatment, especially depending on what it is. Aiding, sure, they already do that with robotics and CT scans and all that, but I want a human there making sure that it’s doing what it’s supposed to. (FG13).*

Participants also noted the uniqueness of each patient, and commented on the resultant individuality required in approaching medical decision making. They viewed the providers’ role in using AI as one of adapting AI recommendations to the patient’s unique personal situation, ensuring that they are not harmed and that patients follow through with clinical recommendations.

*[I]t’s important to take into account that people, depending on what the AI comes out with, people might not be willing to go with what that is, they might need alternates. And also just the question of creativity, like what if the solution were actually something where you would have to think outside the box? … What if it’s something they haven’t encountered before? (FG13).*

### Preservation of patient choice and autonomy

Participants reported that the preservation of choice was an important factor in their overall comfort with applications of AI in healthcare. They felt that patients should have the right to choose to have an AI tool used in their care and be able to opt-out of AI involvement if they felt strongly.

*I think it all comes back to choice, though, I think everybody’s getting the mentality that, and maybe I’m wrong, but that an AI is being pushed, but at the end of the day, our choice is still our choice, and it’s not being taken away. (FG 15).*

In addition to the ability to choose whether an AI tool is used or not, participants wanted to have the ability to dispute the recommendations of an AI algorithm, or correct those recommendations if they believed they were in error. Participants were uncomfortable relying solely on recommendations made by an AI without being able to evaluate the rationale for those recommendations directly themselves.

*So I’d rather know what they’re observing and, if it’s [AI] wrong, I would [want to] be able to correct it rather than have them just collect data and make assumptions. (FG 13).*

### Concerns about healthcare costs and insurance coverage

Participants also voiced concerns that AI tools might increase healthcare costs and that those costs might be passed on to patients. While participants acknowledged that AI might make the delivery of some healthcare services more efficient, they anticipated high development and deployment costs. They felt that adding another advanced technology would likely increase the cost of their healthcare.

*So it sounds expensive, and health care is already fairly expensive. To go on his note, a lot of times you can get something that works just as well for a lot less or you could get something super fancy, that makes you think, hey I got this big fancy thing, but it really doesn’t do any better than the original cheaper version. (FG 9).*

Additionally, participants worried about the impact that AI recommendations could have on what types of treatment their insurance providers would cover. For example, some participants were concerned that an AI algorithm might recommend a treatment that they could not afford. Similarly, others worried that insurance companies might chose to cover only those treatments that are supported by AI recommendations, thereby taking away some of the discretion traditionally reserved for physicians.

*Is insurance only gonna cover what the machine says it is and not look for anything else? There is no reason for further diagnostics because the machine already did it? I mean we already have a situation in our healthcare system where money comes into play for diagnosing things. (FG 9).*

Participants recognized how the ability of AI to draw connections and make highly accurate predictions from images or complex symptoms could be very helpful. However, they were concerned that new types of predictions could result in new forms of discrimination. Participants were especially worried that insurance companies would use AI to discover otherwise unknown medical information that could be used to deny coverage or increase premiums.

*I mean, … that information is wonderful, but who’s gonna get it after the doctors look at it is my big thing. Is the insurance company gonna take it, and now all of a sudden … my premium doubles for health insurance? (FG1).*

### Ensuring data integrity

Participants considered the impact of data quality on AI tools and their recommendations, and had several concerns related to the way healthcare AI might be developed using flawed datasets, potentially resulting in harm to patients. They felt data from the electronic health record was not accurate enough to be reliable in teaching healthcare AI, citing personal experiences with errors they had found in their own health records.

*There’s a lot of discrepancies in the medical record I must say, especially now that you can see your portal. I know I’ve seen things saying that certain things were done or about myself and procedures that were totally not true. So I’ve had a lot of different things in my medical chart that are inaccurate, very inaccurate, so if they’re training an artificial intelligence that this is facts, it’s like, well no. (FG 4).*

Participants were also concerned about the possibility that AI tools might reinforce existing biases in healthcare datasets. They explained that this could happen as a result of an inherently biased learning dataset or from developers unintentionally incorporating their own bias into an AI algorithm.

*Prejudices that people can have, like it could absorb those or it could be taught to work against them, like a lot of people who are overweight have said that their providers assume that that’s the cause and ignore doing other tests or pursuing other avenues, and if an AI wasn’t going to make the assumption that that was what was the problem, then that would be good, but if it was learning from people around it that it should make that assumption, then it would perpetuate the problem. (FG 13).*

### Risks of technology-dependent systems

Participants also expressed concerns about technological systems that might be highly dependent on new AI technologies and worried that some risks might be exacerbated if AI were to be widely deployed in medicine. One such concern was a worry about a systems-level crash or mass technological failure, and the impact this might have on a clinical system that is heavily reliant on AI tools.

*I have some background in electronics, and one thing you can guarantee with electronics is they will fail. Might not be now, might never happen in 10, 20 years. The way things are made, ‘cause I’ve actually worked in the industry of making medical equipment, it’s all about using the cheapest method to get the end result. Well, electronics fail. They just do. (FG9).*

Additionally, participants brought up examples of bad actors hacking into AI systems and manipulating these tools for nefarious purposes.

*I was just gonna say another concern that I think I would have, just because of the way our world is evolving and revolving, is can that artificial intelligence be hacked? Who can control that? …I don’t know. Because any time you have a computerized program, I don’t care what anybody says, it can and it will get hacked because there’s always somebody that’s out there just to do evil rather than good. (FG15).*

These concerns were compounded by the perception that healthcare providers could easily become overly dependent on AI tools, and over time might not be able to provide high-quality care if access to those healthcare AI tools was unavailable.

*If they were to get hacked or a system goes down … like what’s the contingency plan, but what is the contingency plan? If you have all these doctors who are so used to having this artificial intelligence read all these, and they don’t have the skill of reading it, then what happens? (FG6).*

## Discussion

The patients we consulted shared a variety of concerns that will shape their perceptions of future AI applications in healthcare. While they envisioned AI having a generally positive impact on healthcare, this view was contingent on proactive oversight that mitigates potential harms resulting from AI. While participants were able to appreciate the widereaching impact of AI on healthcare, their concerns centered on specific ways in which AI might result in harm to them personally, or to those they care about. This finding contrasts with much of the existing literature examining the potential implications of AI for healthcare, which tends to take a more abstract approach that is not connected to the practical concerns that patients and families may have, such as the potential for AI to limit access to a preferred medication or increase healthcare costs^12,15^. Participants expected to be protected from these harms and felt that physician oversight would be critical. Translational researchers and clinical implementation teams deploying new AI tools must be aware of these expectations if they hope to ensure the successful integration of AI into healthcare systems.

A subset of the concerns voiced by our participants are reminiscent of concerns raised about prior medical advances, including worries about higher costs, discriminatory uses, and fewer choices available to patients and providers. Both personal and national healthcare expenditures have been rising steadily for decades^16^, and many interventions that promise to increase efficiency have not delivered on their promise to decrease healthcare costs^17,18^. Similarly, respect for patient autonomy is one of the core principles of medical ethics^19^, and participants were clear that they wanted to be able to choose whether or not to have AI tools as a component of their care. To the extent that AI tools could operate surreptitiously in the background, patients who do not want AI used in their care may not know it is being used, which could result in significant breaches of public trust should this approach to AI deployment be discovered by those patients. Similarly, while concerns about discrimination based on predictive analytics are reminiscent of concerns about genetic information^20^, it is unclear if legislators will recognize these similarities and develop special protections that apply to AI-enabled medical predictions. Here too, these considerations highlight the value of proactive engagement with patients, both to understand their concerns but also to consider what effective policy responses might entail.

Other patient apprehensions about AI applications in healthcare did not have obvious parallels to concerns about prior healthcare technologies. These concerns mirror some of the most contentious debates about the impact that AI may have on medicine. For example, participants stressed the role of physicians as safety monitors. A similar conversation is reflected in contemporary literature advocating for the necessity of the physician in AI-driven healthcare systems^21–25^. Academics and patients alike seem to feel that the physician must be at the center of medical decision-making to preserve patient safety^22,26^. Participants were also concerned about the future of healthcare systems, as they become increasingly dependent on digital tools. The multiple challenges of ensuring source-data quality, algorithmic reliability, and unbiased AI outcomes have been recognized as a substantial ethical and technological barrier to successful clinical implementation by bioethicists and AI developers alike^15^.

Addressing patient apprehensions about healthcare AI will require creative solutions that incorporate systems-level trust building, proactive technological innovation, and a recognition of the complex social forces at work. Those advocating for clinical uses of AI tools should support the development of transparent oversight mechanisms that promote stakeholder engagement at each step of AI development and implementation—from the curation of healthcare datasets to widespread clinical usage^27^. Importantly, our results show that patients recognize and are beginning to grapple with the many nuanced issues raised by applications of AI in healthcare. Despite the abstractness of machine learning, and lack of personal experience with AI, participants were quickly able to appreciate how they and their families could be impacted (and potentially harmed) by healthcare AI without appropriate oversight. Those concerns could easily evolve into a deep skepticism about the promise of AI without consistent and deliberate patient engagement.

A limitation of this study is the racial and ethnic diversity of our sample, which was limited by our recruitment methods, which involved contacting primary care patients at a large health system in the upper Midwest. Additionally, many participants had personal or familial connections to healthcare occupations, which likely influenced their perceptions of medical innovation. The education level of participants and their insurance coverage was higher than typical, which also may have impacted their engagement with topics related to healthcare. These limitations suggest caution in generalizing the findings we report to other clinical settings and patient populations.

While use of focus-group methods was a strength of the study, allowing us to explore a wide range of patient perspectives on healthcare AI, use of specific case studies to structure focus-group discussions may have influenced our results by encouraging participants to focus on more familiar applications of AI in healthcare. Additionally, the technical complexity of the AI tools we examined may have resulted in inaccurate participant understandings of those technologies and their potential impact.

While this research study provides important insights into patient apprehensions about applications of AI in healthcare, the limitations above highlight a clear need for additional research. Of note, patient-centered research involving underrepresented or historically disadvantaged populations is crucial for ensuring equitable and just applications of AI in healthcare. Additionally, future research studies should seek to characterize the frequency and potential impact of various patient concerns, using survey methods and behavioral models of technology acceptance. Patient engagement around real-world AI tools at various stages of implementation are also critical for ensuring that AI innovation is responsive to patient concerns. Finally, patient perspectives on healthcare AI are only one important factor in the ecosystem of responsible AI development and implementation. Future research should also examine systems-level readiness for healthcare AI and engage other stakeholders, including healthcare providers, payers, and administrators.

Addressing patient concerns relating to AI applications in healthcare is essential for effective clinical implementation. While participants in our study may not have had a complete understanding of AI algorithms or their capabilities, they were able to engage with core concepts relating to healthcare AI and could readily express their expectations and apprehensions. Our results clarify several potential sources of patient concern about applications of AI in healthcare and highlight patients’ desire for physician-led oversight of these technologies. If this expectation is not met, it is possible that we could see a third “AI Winter” in which fears of patient harm lead to widespread rejection of healthcare AI by patients and their providers^9^. To avoid that possibility, it is critical that AI developers engage the public in dialogue about both the potential benefits and harms of applications of AI in healthcare^28^.

## Methods

### Participant recruitment

We contacted 946 patients who visited a Mayo Clinic primary care facility in Minnesota or Wisconsin between November 19, 2019 and February 25, 2020. Potential research participants were recruited by phone. Study inclusion criteria included being conversant in English and being 18 years or older. Participants received $50 for their participation. This study was approved by the Mayo Clinic Institutional Review Board (protocol #19-010128). We did not solicit personal health information from participants, and oral consent was obtained for all participants at the beginning of each focus group before data collection began in accordance with the IRB recommendations.

### Data collection

We selected a focus group design for this study because we anticipated participants having limited prior knowledge of the subject area. Focus groups encourage participants to react to each other’s comments and questions, revealing more nuanced views. Focus groups also allow for a more accessible and open discussion for individuals with varying levels of familiarity with the topics discussed. This approach allowed us to capture diverse opinions generated through the discussion and reflection process^29^.

Focus groups were co-facilitated by three research team members, with one person serving as the primary moderator, and the other two taking field notes and asking follow-up questions to clarify critical points^30,31^. Participants were first asked a series of general questions about AI to gauge their knowledge and familiarity such as “What does AI mean to you” or “What comes to mind when you think about AI and medicine?”. The moderators then offered a brief definition of AI, along with examples of nonmedical applications of AI. The explanation of AI given during the focus group was specific to machine learning, a method of providing data to algorithms without explicit programming, allowing the algorithms to optimize mapping connections between input and output data points^32^. Case studies of specific uses of AI in medicine were then presented to participants for discussion and reflection, followed by questions about participants’ comfort with and concerns about each case study^33^ such as “What are your initial thoughts or reactions to this AI tool?” or “How would you feel about this technology being used for you or your loved one?” Some cases also had specific probes about data use, preferred situations, or relationships with providers. The focus group concluded with a broad discussion of AI applications in healthcare, including general concerns, sources of enthusiasm, and comparisons of perspectives across the different case studies including questions such as “What tradeoffs do you see with AI in healthcare and how do you balance them?”.

The case studies were selected to be representative of emerging applications of AI in healthcare, as determined by a review of the literature on AI technologies^2–4^. Each case was based on a specific type of tool, which allowed participants to engage with the tools in a way that was both generalizable but grounded in reality. Focus groups 1–6 used three case studies: an image analysis tool, a ChatBot for asking questions about a medical procedure, and a risk prediction tool that analyzed and flagged patients at risk of developing a preventable condition. (These case studies are available from the authors upon request.) Focus groups 7–15 used three different cases: a complex diagnostic tool, a well-person visit involving a ChatBot, and a tool for monitoring patients in an intensive care unit (ICU). After each focus group, the research team met to debrief about the session. Each team member generated a field note summarizing the discussion and describing general themes. A fourth team member then combined the memos from the three team members who were present at the focus group to generate a consensus memo for that focus group^34,35^. The moderator guide was also refined throughout data collection to improve its clarity and effectiveness.

Participants were asked to complete a short questionnaire at the beginning of the focus group to collect demographic data, which included: age, gender, race, ethnicity, and educational attainment. The questionnaire also included two questions related to participant’s work experiences; one question examined work experience in a technology or computer-science field, and a second question examined experience working in healthcare or the health sciences. These questions were included to assess the extent to which our sample included persons whose work might be impacted more directly by applications of AI in healthcare, which we hypothesized would impact participants’ familiarity and engagement with the topics discussed.

### Data analysis

After the first six focus groups, a synthesized memo was generated for each of the cases, summarizing preliminary findings and emerging themes^34,35^. A second set of case studies was designed using this preliminary analysis, resulting in three completely new cases. The same memoing process was done for the second set of case studies after the remaining focus groups were completed. Data collection, memoing, and preliminary data analysis continued until the study team agreed that thematic saturation had been reached^36^. Each focus group was audio recorded, with recordings sent to a transcription service and transcribed verbatim. All recordings were deidentified and reviewed for accuracy by the study team.

Transcripts were analyzed using a modified inductive approach which employed constant comparison analysis^37–39^. The qualitative analysis team consisted of one primary coder (J.P.R.) and two secondary coders (C.S., S.C.). A preliminary codebook was generated using the memos that were written and synthesized during data collection^34^. All three analysis team members then applied the codebook to three transcripts from different points in data collection, revised the codebook based on those experiences, and generated a final codebook that was used to examine the entire dataset^40^. Each transcript was coded independently by two coders. The two coders then met to discuss code any discrepancies until consensus was reached. All coding was done using NVivo 11 Software. Data available upon request.

### Reporting summary

Further information on research design is available in the Nature Research Reporting Summary linked to this article.

## Supplementary information

Reporting Summary

## Data Availability

Additional supporting data available upon request to authors
