# Supplementary material for: Patient apprehensions about the use of artificial intelligence in healthcare
Source: NPJ Digit Med. 2021 Sep 21;4:140. doi: 10.1038/s41746-021-00509-1 (PMC8455556; doi:10.1038/s41746-021-00509-1)
Supplement: Supplementary file 1 — Reporting Summary [file 41746_2021_509_MOESM1_ESM.pdf]

nature portfolio

Corresponding author(s): Richard Sharp

Last updated by author(s): Aug 16, 2021

Reporting Summary

Nature Portfolio wishes to improve the reproducibility of the work that we publish. This form provides structure for consistency and transparency in reporting. For further information on Nature Portfolio policies, see our Editorial Policies and the Editorial Policy Checklist.

Statistics

For all statistical analyses, confirm that the following items are present in the figure legend, table legend, main text, or Methods section.

n/a	Confirmed	
☐	☒	The exact sample size (n) for each experimental group/condition, given as a discrete number and unit of measurement
☒	☐	A statement on whether measurements were taken from distinct samples or whether the same sample was measured repeatedly
☒	☐	The statistical test(s) used AND whether they are one- or two-sided<br>*Only common tests should be described solely by name; describe more complex techniques in the Methods section.*
☒	☐	A description of all covariates tested
☒	☐	A description of any assumptions or corrections, such as tests of normality and adjustment for multiple comparisons
☒	☐	A full description of the statistical parameters including central tendency (e.g. means) or other basic estimates (e.g. regression coefficient) AND variation (e.g. standard deviation) or associated estimates of uncertainty (e.g. confidence intervals)
☒	☐	For null hypothesis testing, the test statistic (e.g. F, t, r) with confidence intervals, effect sizes, degrees of freedom and P value noted<br>*Give P values as exact values whenever suitable.*
☒	☐	For Bayesian analysis, information on the choice of priors and Markov chain Monte Carlo settings
☒	☐	For hierarchical and complex designs, identification of the appropriate level for tests and full reporting of outcomes
☒	☐	Estimates of effect sizes (e.g. Cohen's d, Pearson's r), indicating how they were calculated

Our web collection on statistics for biologists contains articles on many of the points above.

Software and code

Policy information about availability of computer code

Data collection	*Provide a description of all commercial, open source and custom code used to collect the data in this study, specifying the version used OR state that no software was used.*
Data analysis	Data was analyzed using NVivo 11 Software

For manuscripts utilizing custom algorithms or software that are central to the research but not yet described in published literature, software must be made available to editors and reviewers. We strongly encourage code deposition in a community repository (e.g. GitHub). See the Nature Portfolio guidelines for submitting code & software for further information.

Data

Policy information about availability of data

All manuscripts must include a data availability statement. This statement should provide the following information, where applicable:

- Accession codes, unique identifiers, or web links for publicly available datasets
- A description of any restrictions on data availability
- For clinical datasets or third party data, please ensure that the statement adheres to our policy

Additional data is available upon request

Field-specific reporting

Please select the one below that is the best fit for your research. If you are not sure, read the appropriate sections before making your selection.

☐ Life sciences ☒ Behavioural & social sciences ☐ Ecological, evolutionary & environmental sciences

For a reference copy of the document with all sections, see nature.com/documents/nr-reporting-summary-flat.pdf

Behavioural & social sciences study design

All studies must disclose on these points even when the disclosure is negative.

Study description	This study is a qualitative focus group study with a supplemental demographics survey.
Research sample	Participants included people who receive primary care at Mayo Clinic or a Mayo Clinic Health System location in Rochester MN, Mankato MN, Eau Claire WI, and La Crosse WI. The average age of participants was 53.5 years old and 49.4% female. This sample was chosen to be representative of the local population.
Sampling strategy	A full list of patients who received primary care at one of the selected locations was pulled and stratified by age group and gender. The research team then called patients, alternating gender and age group, until each focus group had 10 individuals who agreed to participate. The research team continued to conduct focus groups, making modifications to moderator guide probes, until thematic saturation was achieved.
Data collection	Data was collected during the focus group via direct audio recording by an encrypted Olympus recorder. The three members of the research team also took notes using pen and paper. No-one was present aside from the three research team members and participants.
Timing	Patients were contacted and focus groups were performed between November of 2019 and February of 2020.
Data exclusions	No data was excluded from primary analysis. Given the quantity of data from 15 focus groups, the authors chose to split the data into multiple, thematically distinct, secondary analyses.
Non-participation	15.8% of contacted patients who were contacted agreed to participate in the study. 58% of patients who agreed to participate actually participated in the focus groups. Patients who were contacted but did not participate were either not interested in participation, were not able to come at the time the focus group was being offered, or did not answer their phone or call the research team back.
Randomization	Participants were allocated into focus groups based on their availability, and all focus group data was analyzed together.

Reporting for specific materials, systems and methods

We require information from authors about some types of materials, experimental systems and methods used in many studies. Here, indicate whether each material, system or method listed is relevant to your study. If you are not sure if a list item applies to your research, read the appropriate section before selecting a response.

Materials & experimental systems

n/a	Involved in the study	
☒	☐	Antibodies
☒	☐	Eukaryotic cell lines
☒	☐	Palaeontology and archaeology
☒	☐	Animals and other organisms
☐	☒	Human research participants
☒	☐	Clinical data
☒	☐	Dual use research of concern

Methods

n/a	Involved in the study	
☒	☐	ChIP-seq
☒	☐	Flow cytometry
☒	☐	MRI-based neuroimaging

Human research participants

Policy information about studies involving human research participants

Population characteristics	See above
Recruitment	Participants were contacted by phone call because they received primary care at Mayo Clinic. There is possible self-selection bias for people interested in participating in biomedical research or participants who are interested in artificial intelligence.
Ethics oversight	This study was approved by the Mayo Clinic Institutional Review Board

Note that full information on the approval of the study protocol must also be provided in the manuscript.
